# Diagnostic Assay for *Rickettsia japonica*

**DOI:** 10.3201/eid1512.090252

**Published:** 2009-12

**Authors:** Nozomu Hanaoka, Minenosuke Matsutani, Hiroki Kawabata, Seigo Yamamoto, Hiromi Fujita, Akiko Sakata, Yoshinao Azuma, Motohiko Ogawa, Ai Takano, Haruo Watanabe, Toshio Kishimoto, Mutsunori Shirai, Ichiro Kurane, Shuji Ando

**Affiliations:** National Institute of Infectious Diseases, Tokyo, Japan (N. Hanaoka, H. Kawabata, A. Sakata, M. Ogawa, A. Takano, H. Watanabe, T. Kishimoto, I. Kurane, S. Ando); Yamaguchi University School of Medicine, Yamaguchi, Japan (M. Matsutani, Y. Azuma, M. Shirai); Miyazaki Prefectural Institute for Public Health and Environment, Miyazaki, Japan (S. Yamamoto); Ohara General Hospital, Fukushima, Japan (H. Fujita); Gifu University, Gifu, Japan (H. Kawabata, A. Takano, H. Watanabe)

**Keywords:** Rickettsia japonica, specific ORF, real-time PCR, diagnosis, genome, bacteria, dispatch

## Abstract

We developed a specific and rapid detection system for *Rickettsia japonica* and *R. heilongjiangensis*, the causative agents of spotted fever, using a TaqMan minor groove binder probe for a particular open reading frame (ORF) identified by the *R. japonica* genome project. The target ORF was present only in *R. japonica*–related strains.

*Rickettsia*, a genus that includes the causative agents for spotted fever rickettsioses and typhus fever, comprises obligate intracellular bacteria (*1*). The first case of Japanese spotted fever (JSF), caused by *R. japonica* (*2*), was reported in 1984 in Japan (*3*). According to the national surveillance system in Japan (http://idsc.nih.go.jp/idwr/ybata/report-Ea.html), JSF cases, including sporadic cases resulting in death, have been gradually increasing. Rapid diagnosis of rickettsial infections is important because rickettsioses can be cured when appropriate antimicrobial drug treatment is given during the early clinical stages of the disease. Furthermore, development of a rapid and specific diagnostic system for *R. japonica* is now a matter of increasing urgency (*4*) because JSF has also been reported in several other countries in Asia (*1*).

## The Study

Since 1998, thirteen complete *Rickettsia* genome sequences have been reported (www.ncbi.nlm.nih.gov/Genbank/index.html). In addition, the National Center for Biotechnology Information (NCBI) genome project for *R. japonica* strain YH has recently concluded (project ID 38487). Results of this project show specific DNA regions for *R. japonica* in the *Rickettsia* genome. One of these regions includes a 216-bp open reading frame (ORF) (GenBank accession no. AB437281). On the basis of information from this genome project, we performed DNA sequencing for this 216-bp ORF to determine whether the specific DNA sequences are conserved in all *R. japonica* strains and other closely related strains, including *R. heilongjiangensis* (*5*) and *Rickettsia* sp. LON (*6*).

*R. heilongjiangensis* is also a causative agent of spotted fever in northeastern Asia and has been classified within the *R. japonica* group (*5*). Several studies have reported that *Rickettsia.* sp. LON strains also have similar sequences to *R. japonica.* Our PCR can easily distinguish *Rickettsia* sp. LON strains (LON-2, LON-9, and LON-13) from *R. japonica* strains. This test can help in the diagnosis because *Rickettsia* sp. LON strains have only been isolated from ticks and may not be pathogenic in humans (*6*).

DNA sequencing was performed by using an ABI PRISM BigDye Terminator version 3.1 Kit (Applied Biosystems, Foster City, CA, USA) with an ABI 3130 sequence detector. DNA sequences were aligned by ClustalW software (http://clustalw.ddbj.nig.ac.jp/top-e.html) with an open gap penalty of 15, a gap extension penalty of 6.66, a gap distance of 8, and a maximum division penalty of 40. For determination of the DNA sequence for the 216-bp ORF, the primer pair of JapoSP5′ (5′-ACAACATCAATATTATAATTAGTATCC-3′) and JapoSP3′ (5′-TTCACGTATGTCTATATATGCTGCAGCG-3′) was used to amplify a 564-bp section, including this ORF, because this unique DNA sequence was located as the inserted sequence of the homolog for *R. conorii* RC1338 (Figure, panel A).

**Figure:**
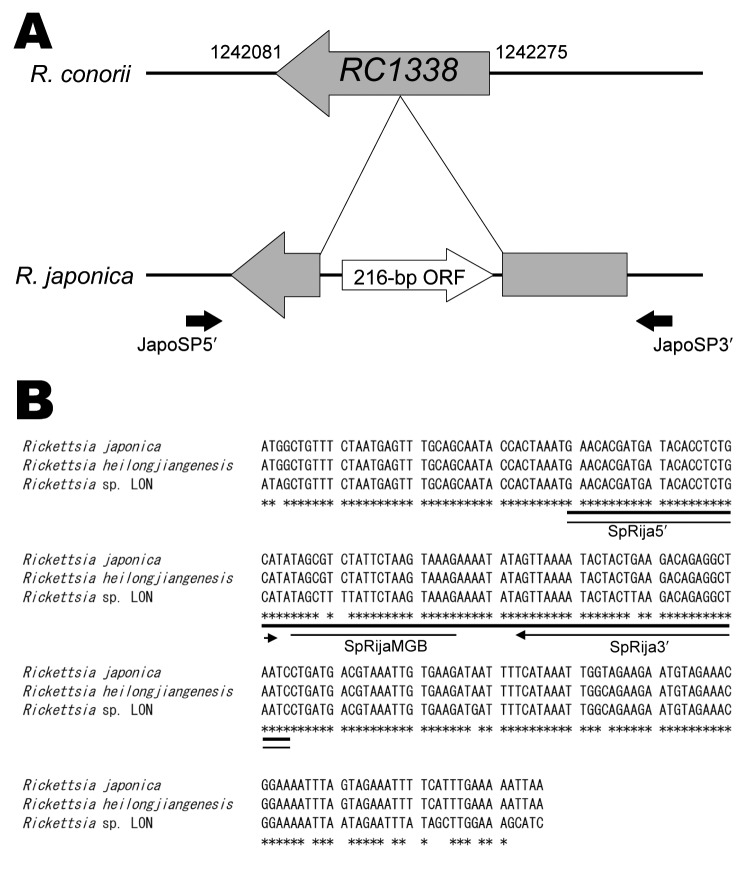
Unique DNA sequence in the *Rickettsia japonica* genome that analyzed PCR in this study. A) Comparative genome map of the 216-bp open reading frame (ORF). The *R. japonica*–specific sequence region (AB437281) in the *R. japonica* genome and the complete genome sequence of *R. conorii* strain Malish 7 were compared. The *RC1338* DNA sequence and the mapping position data for *R. conorii* were obtained from the *Rickettsia* genome database (www.igs.cnrs-mrs.fr/mgdb/Rickettsia). Two solid black arrows indicate primer positions; this region was amplified and sequenced with the same primers. B) Alignments of *R. japonica*–specific 216-bp ORF (AB437281) between *R. japonica* YH, *R. heilongjiangensis* CH8-1, and *Rickettsia* sp. LON, as performed by the program ClustalW (www.ebi.ac.uk/clustalw), and the positions of primers and probe in real-time PCR. Primer positions and directions (black arrows) and the TaqMan minor groove binder (MGB) (line) probe position are shown. The 216-bp ORF of *R. heilongjiangensis* and *Rickettsia* sp. LON were registered on GenBank with accession nos. AB512783 and AB512784, respectively. DNA sequences are identical among *R. japonica* strains and *Rickettsia* sp. LON strains (asterisks). The alignment was edited with BioEdit version 7.0.0 (www.mbio.ncsu.edu/BioEdit/bioedit.html).

The nucleotide sequence of this ORF was identical among 5 of the *R. japonica* strains: DT-1, YH, FLA-1, HH-8, and HH-9 (100%); the sequence was highly conserved with significant identity in *R. heilongjiangensis* (99.5%), except for the *Rickettsia* sp. LON strains (92.1%; Figure, panel B). This 216-bp ORF had been previously applied to BLAST searches with NCBI blast (nblast) for humans, mice, and others (http://blast.ncbi.nlm.nih.gov/Blast.cgi). The results showed that no similar ORF had been reported to date. Therefore, we focused on this conserved region of the 216-bp ORF to develop a TaqMan minor groove binder (MGB) probe (Applied Biosystems) that could detect the pathogenic *R. japonica* group, including *R. heilongjiangensis*, with a high degree of specificity.

Oligonucleotide primers (SpRija5′ and SPRija3′) and the TaqMan MGB probe (SpRijaMGB) were designed by using Primer Express software version 2.0 (Applied Biosystems Figure, panel B). The detection probe was labeled with the fluorescent reporter FAM (carboxyfluorescein labeling) at the 5′ end; the nonfluorescent quencher and MGB were labeled at the 3′ end (Figure, panel B). The detection sequence (shown as a bold line in Figure, panel B) was registered in GenBank (accession no. AB437281).

Real-time PCR was performed by using an ABI 7500 system (Applied Biosystems). DNA polymerase (perfect real-time PCR) for the PCR was obtained from Takara Bio (Kyoto, Japan). A 20-μL sample was added to each well of a 96-well microplate (Thermo Fisher Scientific Inc., Waltham, MA, USA) according to the manufacturer’s instructions. Thermal cycle protocol was performed as follows: first incubation stage, 20 s at 95°C; second stage, 5 s at 95°C and 34 s at 60°C. The second stage was repeated 45 times. For analysis of real-time PCR, the threshold line was fixed at 0.2 to avoid detection of nonspecific fluorescence. This detection procedure can be completed within 1 h.

The reactivity of this assay was examined by using various copy numbers of synthetic *R. japonica* DNA fragments that were amplified by the primer pairs JapoSP5′ and JapoSP3′ within the *R. japonica* genome. Genomic DNA of *R. japonica* strain YH was prepared from cultivated bacteria according to methods proposed by Furuya et al. (*7*). A calibration curve was generated with 5 calibrators, ranging from 10^2^ to 10^9^ copies/well in triplicate. We found a linear correlation (R>0.99) between the detection cycle numbers and *R. japonica* DNA copy numbers from 10^2^ to 10^9^ copies/reaction (data not shown).

A total of 26 rickettsial strains, classified into 11 species, were used in this study (Table 1). Specificities for this TaqMan PCR are also summarized in Table 1. Genomic DNA of *R. prowazekii* and *R. rickettsii* were prepared from antigen slides (Panbio Inc., Sinnamon Park, Queensland, Australia) by using a Gentra Puregene kit (QIAGEN, Valencia, CA, USA). Genomic DNA of other *Rickettsia* strains was also prepared from cultivated bacteria according to methods proposed by Furuya et al. (*7*).

**Table 1:** Reactivity of the real-time PCR for *Rickettsia* strains used in this study*

Species	Strain	Isolation source	Reference	Real-time PCR
*Rickettsia asiatica*	IO-1	*Ixodes ovatus*	ATCC VR-1593 (*8*)	–
	IO-2	*I. ovatus*	(*8*)	–
	IO-3	*I. ovatus*	(*8*)	–
	IO-25	*I. ovatus*	(*8*)	–
	IO-38	*I. ovatus*	(*8*)	–
*R. conorii*	Malish 7	Human	ATCC VR 613T	–
*R. heilongjiangensis*	CH8-1	*Haemaphysalis concinna*	(*6*)	+
*R. helvetica*	IM-1	*Ixodes monospinosus*	(*9*)	–
	IP-1	*I. persulcatus*	(*9*)	–
	IP-2	*I. persulcatus*	(*10*)	–
	IP-6	*I. persulcatus*	This study	–
*R. honei*	TT-118	*Ixodes* sp.	(*11*)	–
*R. japonica*	DT-1	*Dermacentor taiwanensis*	(*12*)	+
	YH	Human	ATCC VR-1363	+
	FLA-1	*Haemaphysalis flava*	(*9*)	+
	HH-8	*H. hystricis*	This study	+
	HH-9	*H. hystricis*	This study	+
*R. prowazekii*	breinl	Human	(*13*)	–
*R. rickettsii*	Sheila Smith	Human	(*14*)	–
*R. sibirica*	246	Human	ATCC VR-151	–
*R. tamurae*	AT-1	*Amblyomma testudinarium*	ATCC VR-1594 (*12*)	–
	AT-4	*A. testudinarium*	(*6*)	–
	AT-13	*A. testudinarium*	(*6*)	–
*R. typhi*	Wilmington	Human	ATCC VR-144	–
*Rickettsia* sp. LON	LON-2	*Haemaphysalis longicornis*	(*6*)	–
	LON-9	*H. longicornis*	(*6*)	–
	LON-13	*H. longicornis*	(*6*)	–

*+, positive; –, not detected.

Our results showed that this novel assay could detect all 5 *R. japonica* strains and 1 *R. heilongjiangensis* strain used in this study. However, it could not detect *R. rickettsii*, *R. prowazekii*, or other *Rickettsia* strains. These results indicate that the combination of probes and primers in this study had high specificity for the pathogenic *R. japonica* group. Nonspecific reactions were not observed when genomic DNA from human or murine fibroblasts was used in any of the assays (data not shown).

The detection limits of this PCR were compared to those of conventional PCRs by using serially diluted genomic DNA. The conventional PCRs, designated as Rj5-Rj10 and R1-R2 assays, were designed to detect the 17-kDa antigen gene of *Rickettsia*, by using a primer set of Rj5 (5′-CGCCATTCTACGTTACTACC-3′) and Rj10 (5′-ATTCTAAAAACCATATACTG-3′) (*7*) and R1 (5′-TCAATTCACAACTTGCCATT-3′) and R2 (5′-TTTACAAAATTCTAAAAACC-3′) (*15*), respectively. Since 1996, these assays have been used for molecular diagnosis of JSF in clinical laboratories in Japan. Recent studies have suggested that a TaqMan PCR assay may be as much as 100× more sensitive than these assays (data not shown). Therefore, in our study, the TaqMan PCR was assumed to be much more sensitive than the conventional assays that are known to show false-negative results, even for a patient with acute-stage JSF.

We requested clinical samples to verify the validity of our assay. Eighteen DNA templates were extracted from blood clots collected from 18 patients in the acute stages of illness (male:female ratio 1:1; average age 64.1 years [range 27–88 years); average number of days after onset of fever 4.6 [range 2–7 days; Table 2]). These templates were reexamined by using our TaqMan PCR. Although the conventional assays could not detect the presence of any *Rickettsia* DNA, 9 of 18 samples displayed positive results with the TaqMan PCR (Table 2). The blood clot from the patient in whom Scrub typhus disease was previously diagnosed was used as a negative control in this real-time PCR assay, resulted were not detected. Our TaqMan PCR is currently available to clinical laboratories that need to rule out false-negative results in molecular diagnoses.

**Table 2:** Application of PCRs for blood clot specimens derived from acute-stage Japanese spotted fever patients*

Patient no.	Age, y/sex	Days after onset of fever†	Laboratory examinations				Real-time PCR¶
			*Rickettsia* isolation‡	Serodiagnosis§	Conventional PCR		
					Rj5–Rj10 assay	R1–R2 assay	
C1	83/F	5	+	+	**–**	**–**	38.2 ± 0.6
C2	35/F	6	+	+	**–**	**–**	–
C3	70/M	6	+	+	**–**	**–**	37.6 ± 1.3
C4	71/M	3	+	+	**–**	**–**	–
C5	66/F	6	+	+	**–**	**–**	38.4 ± 1.0
C6	49/M	3	NT	+	**–**	**–**	–
C7	88/F	5	NT	+	**–**	**–**	40.7 ± 0.4
C8	49/M	7	NT	+	**–**	**–**	31.4 ± 1.1
C9	65/F	3	NT	+	**–**	**–**	36.0 ± 0.5
C10	78/M	4	NT	+	**–**	**–**	39.0 ± 0.8
C11	72/F	4	NT	+	**–**	**–**	–
C12	68/F	5	NT	+	**–**	**–**	–
C13	27/M	5	NT	+	**–**	**–**	–
C14	45/M	7	NT	+	**–**	**–**	–
C15	76/F	5	NT	+	**–**	**–**	39.0 ± 0.5
C16	79/M	3	NT	+	**–**	**–**	39.9 ± 1.0
C17	69/M	2	NT	+	**–**	**–**	**–**
C18	64/F	3	NT	+	**–**	**–**	**–**

*+, positive; NT, not tested; –, not detected. †Blood clot was prepared from whole blood at indicated days after onset of fever. ‡Isolation from whole blood specimens. §Seroconversion was identified by the indirect immunofluorescent test with paired serum specimens. ¶Cycle threshold values are given as means ± standard errors of the means for 3 independent assays.

## Conclusions

JSF is a threat to public health in Japan. Our results suggest that an *R. japonica*–specific 216-bp ORF may have been conserved throughout the *R. japonica* species and closely related *Rickettsia* spp. The newly developed real-time PCR system, which demonstrated a high level of sensitivity and specificity, may be a useful tool for laboratory diagnosis.
